# Two Novel Negative-Sense RNA Viruses Infecting Grapevine Are Members of a Newly Proposed Genus within the Family Phenuiviridae

**DOI:** 10.3390/v11080685

**Published:** 2019-07-26

**Authors:** Alfredo Diaz-Lara, Beatriz Navarro, Francesco Di Serio, Kristian Stevens, Min Sook Hwang, Joshua Kohl, Sandra Thuy Vu, Bryce W. Falk, Deborah Golino, Maher Al Rwahnih

**Affiliations:** 1 Department of Plant Pathology, University of California-Davis, Davis, CA 95616, USA; 2 Istituto per la Protezione Sostenibile delle Piante, Consiglio Nazionale delle Ricerche, 70126 Bari, Italy; 3 Department of Evolution and Ecology, University of California-Davis, Davis, CA 95616, USA; 4 Foundation Plant Services, University of California-Davis, Davis, CA 95616, USA

**Keywords:** grapevine, high throughput sequencing, nsRNA viruses, phylogenetics, *Phenuiviridae*, detection, transmission, evolution

## Abstract

Two novel negative-stranded (ns)RNA viruses were identified by high throughput sequencing in grapevine. The genomes of both viruses, named grapevine Muscat rose virus (GMRV) and grapevine Garan dmak virus (GGDV), comprise three segments with each containing a unique gene. Based on sequence identity and presence of typical domains/motifs, the proteins encoded by the two viruses were predicted to be: RNA-dependent RNA polymerase (RdRp), nucleocapsid protein (NP), and putative movement protein (MP). These proteins showed the highest identities with orthologs in the recently discovered apple rubbery wood viruses 1 and 2, members of a tentative genus (Rubodvirus) within the family *Phenuiviridae*. The three segments of GMRV and GGDV share almost identical sequences at their 5′ and 3′ termini, which are also complementary to each other and may form a panhandle structure. Phylogenetics based on RdRp, NP and MP placed GMRV and GGDV in the same cluster with rubodviruses. Grapevine collections were screened for the presence of both novel viruses via RT-PCR, identifying infected plants. GMRV and GGDV were successfully graft-transmitted, thus, they are the first nsRNA viruses identified and transmitted in grapevine. Lastly, different evolutionary scenarios of nsRNA viruses are discussed.

## 1. Introduction

The phylum *Negarnaviricota*, composed of viruses with negative-stranded (ns)RNA genome, includes species characterized by (i) non-segmented or segmented genomes, (ii) the presence or absence of a membrane enveloping the capsid, and (iii) a diverse host range including plants and animals [1,2]. Examples of nsRNA viruses associated with economically important diseases in plants are rose rosette virus [3], rice stripe virus (RSV) [4], citrus psorosis virus [5], and blueberry mosaic associated virus [6].

Historically, only a relatively small number of nsRNA viruses infecting plants as their primary host have been reported [7]. Recently, however, more novel viruses infecting plants have been discovered around the world. In the last few years, the use of high throughput sequencing (HTS) technology has allowed the identification and characterization of new nsRNA viruses in pistachio [8], citrus [9,10], watermelon [11], and apple [12]. Interestingly, most of these novel nsRNA viruses were classified under the family *Phenuiviridae* (order *Bunyavirales*). To date, there are fifteen recognized genera integrating the family *Phenuiviridae* (https://talk.ictvonline.org/taxonomy): *Banyangvirus*, *Beidivirus*, *Goukovirus*, *Horwuvirus*, *Hudivirus*, *Hudovirus, Kabutovirus*, *Laulavirus*, *Mobuvirus*, *Phasivirus*, *Phlebovirus*, *Pidchovirus*, *Tenuivirus*, *Wenrivirus,* and *Wubeivirus.* Except for members of the genus *Tenuivirus* that are plant-infecting viruses [13], the members of the other genera infect vertebrates, including humans, and arthropods. Viruses belonging to the recently established genus *Coguvirus* also infect plants and, although this genus has not been included yet in the family *Phenuiviridae,* many structural and molecular features and phylogenetic relationships with other phenuiviruses support its classification in this family [9,10].

Grapevine (*Vitis vinifera*) is the crop associated with the highest number of intracellular pathogens [14]. With respect to nsRNA viruses, there is only one preliminary report of tomato spotted wilt virus (TSWV; genus *Orthotospovirus*, family *Tospoviridae*) in this host [15]; however, later studies failed to transmit TSWV to grapevine [16]. During the screening of two selections of Garan dmak and Muscat rose grapevines via HTS, partial sequences with homology to apple rubbery wood viruses 1 and 2 (ARWV1 and ARWV2) were identified. ARWV1 and ARWV2 are two novel nsRNA viruses with tri-segmented genomes recently discovered in apple trees displaying rubbery wood symptoms in Canada [12]. Based on sequence identity and phylogenetic analysis, ARWV1 and ARWV2 were proposed to be representative members of a new genus, tentatively named Rubodvirus, within the family *Phenuiviridae*.

Here, we report the natural occurrence and discovery of two novel nsRNA viruses, named grapevine Muscat rose virus (GMRV) and grapevine Garan dmak virus (GGDV), in grapevine plants. Even more, this is the first evidence for the natural occurrence of phenui-like viruses in grapevine. Later, reverse transcription PCR (RT-PCR)-based assays for the specific detection of both viruses were designed to investigate the prevalence in different grapevine populations. Phylogenetics and transmission of GMRV and GGDV were also investigated.

## 2. Materials and Methods

### 2.1. Plant Material and Total Nucleic Acid Extraction

In 2015, a new selection of grapevine, cultivar Garan dmak, was received as dormant cuttings from Armenia under a USDA Animal and Plant Health Inspection Service (APHIS) controlled import permit for inclusion in the Foundation Plant Services (FPS) collection. Cuttings were propagated under mist and later transferred to single pots; all this within an insect-proof greenhouse enclosure. Six months after bud break, leaf tissue from the Garan dmak plant was collected for HTS as part of the plant certification program at FPS. Additionally, a Muscat rose grapevine at the USDA National Clonal Germplasm Repository (NCGR) but originated from Argentina was sampled and analyzed by HTS during a separate study about the genetic diversity of grapevine leafroll-associated virus 3 (GLRaV-3) [17]. At the time of sampling, both grapevine cultivars did not display any known symptom associated with virus infection.

Total nucleic acid (TNA) extracts were prepared using a MagMax Plant RNA Isolation kit (ThermoFisher Scientific, Sunnyvale, CA, USA) as per manufacturer’s protocol, but adjusting the amount of plant material (leaf petioles) based on the following molecular analysis. For RT-PCR, 0.2 g of tissue was homogenized in 2 mL of guanidine isothiocyanate lysis buffer (4 M guanidine isothiocyanate; 0.2 M sodium acetate, pH 5.0; 2 mM EDTA; 2.5% (*w*/*v*) PVP-40) using a Homex grinder. In the case of HTS, 0.7 g of tissue was homogenized in 7 mL of guanidine isothiocyanate lysis buffer. Subsequently, the quality of TNA extracts was verified using an 18S ribosomal RNA (rRNA) assay [18].

### 2.2. High Throughput Sequencing and Completion of Full Genomes

HTS analysis was performed as described by Al Rwahnih et al. [19]. Briefly, aliquots of TNA from source samples were subjected to rRNA depletion and complementary DNA (cDNA) library construction. Later, cDNA libraries were sequenced using the Illumina NextSeq 500 platform using a single-end 75-bp regime. Illumina reads were demultiplexed and adapter trimmed prior to analysis using Illumina bcl2fastq v2.20.0.422. Trimmed reads were subsequently *de novo* assembled into contigs using SPAdes v3.13 [20]. Generated contigs were compared against the viral database of the National Center for Biotechnology Information (NCBI) using tBLASTx. Novel virus contigs were initially identified by their conserved protein domains. Open reading frames (ORFs) were annotated using HMMER v3.1 [21] to look for Pfam [22] protein domains associated with viruses infecting land plants. In the case of *Phenuiviridae*-like contigs, the large RNAs (RNA 1) were annotated with the bunyavirus RNA-dependent RNA polymerase (RdRp) domain. The medium RNAs (RNA 2) were annotated with the viral movement protein (MP) domain. The small RNAs (RNA 3) were annotated with the *Tenuivirus/Phlebovirus* nucleocapsid protein (NP) domain. The association between the small and the medium RNAs was investigated by BLASTn sequence similarity of their 5′ ends. These contig sequences were subsequently confirmed by BLASTx hits to the NCBI nucleotide (nt) database which produced top hits to different accessions of ARWV2 and ARWV1.

To complete the 5′ end of each RNA segment present in GMRV and GGDV, the SMARTer RACE 5′/3′ Kit (Takara Bio Inc., Mountain View, CA, USA) was employed following the instructions provided by the manufacturer. In the case of the 3′ ends, the methodology described by Navarro et al. [9] was used, which involved the addition of a poly(A) tail to the RNA template.

### 2.3. Accession Numbers

GMRV RNA 1, RNA 2, and RNA 3 have the GenBank accession numbers MK728654, MK728655, and MK728656, respectively. GGDV RNA 1, RNA 2, and RNA 3 have the GenBank accession numbers MK728657, MK728658, and MK728659, respectively.

### 2.4. Genome Analysis

Potential ORFs and proteins similar to those encoded by GGDV and GMRV were identified by HMMER v3.1 [21] and BLASTx analysis. Conserved domains present in the putative proteins were searched in the Pfam [22] database. When indicated, alignments of sequences and/or structures were performed with PROMALS3D [23]. Phyre2 web portal [24] was used to identify the best template for modeling the tertiary structure of the putative NP of both GGDV and GMRV.

### 2.5. Phylogenetic Analyses

Phylogenetic trees of genomic segments present in nsRNA viruses, including the core (signature domains) amino acid (aa) sequence of RdRp and the complete aa sequences of MP and NP, were built using MEGAX [25] from multiple alignments generated by COBALT [26]. The phylograms were inferred adopting the Maximum Likelihood (ML) method with 500 bootstrap replicates. TrimAl [27] was used to remove poorly aligned regions in the RdRp alignment before tree generation. The best-fit aa substitution models (LG+G for RdRp, and LG+G+F for MP and NP) were determined using MEGAX. Information about analysed viruses and corresponding GenBank accessions can be found in the phylograms.

### 2.6. Detection, Survey, and Biological Assay

Four different one-step RT-PCR assays were developed to test plant material for the presence of GMRV and GGDV. Thus, specific primers (Table S1) were designed using Geneious 10.2.6 [28] to amplify fragments in the RdRp and NP genes of each virus. RT-PCR reactions (25 µL volume) included 2 µL of TNA and final primer concentration of 500 nM. These reactions were performed with SuperScript II Reverse Transcriptase (Invitrogen, Carlsbad, CA, USA) and GoTaq Flexi DNA polymerase (Promega, Madison, WI, USA). Thermocycler conditions were 30 min at 52 °C, 35 cycles of 30 s at 94 °C, 45 s at 53 °C, 1 min at 72 °C, and a final elongation step of 7 min at 72 °C. Subsequently, samples tested positive were direct sequenced in both directions to confirm specificity.

A total of 1732 samples collected in three different grapevine populations were screened for GMRV and GGDV using the above-described assays. The NCGR (1206 samples), located in Winters, California, includes selections of table and wine grapevines originated from around the world [29]; the Davis Virus Collection (DVC) (109 samples), which is integrated by plants infected with different viruses [30]; and the FPS pipeline (417 samples) of foreign and domestic introductions. The last two mentioned collections belonging to the University of California-Davis.

To determine the transmissibility of GMRV and GGDV, plants infected by these viruses were used as an inoculum source in a biological assay. Following the methodology described by Al Rwahnih et al. [31], bud chips from the Garan dmak and Muscat rose grapevines were grafted onto Cabernet franc (*V. vinifera*) indicator plants (six replicates per source plant). Grafted plants were maintained in an insect-proof greenhouse for one month to allow the graft to heal before planting in the field. One year later, indicator plants were sampled for virus detection. Likewise, non-graft Cabernet franc plants were used as negative controls.

## 3. Results

### 3.1. Identification of Two Novel nsRNA Viruses Infecting Grapevine by HTS

Analysis of HTS data (Table S2) generated from the transcriptome of two asymptomatic grapevine selections revealed the presence of different viruses and viroids. Thus, the Garan dmak grapevine was found infected by grapevine rupestris stem pitting-associated virus (GRSPaV) (fifteen contigs; length: 315–7686 nts; coverage: 4–20 X), Australian grapevine viroid (AGVd) (one contig; length: 374 nts; coverage: 216 X), grapevine yellow speckle viroid 1 (GYSVd-1) (one contig; length: 321 nts; coverage: 257 X), and hop stunt viroid (HSVd) (one contig; length: 352 nts; coverage: 259 X). In the Muscat rose plant, grapevine virus A (sixteen contigs; length: 206–2785 nts; coverage: 9–382 X), grapevine virus E (seventeen contigs; length: 211–7546 nts; coverage: 60–260 X), grapevine leafroll-associated virus 4 strain 5 (seven contigs; length: 219–3824 nts; coverage: 7–542 X), GLRaV-3 (two contigs; length: 6045-12,412 nts; coverage: 18–44 X) and HSVd (one contig; length: 249 nts; coverage: 493 X) were identified. In addition, six *de novo* contigs (length: 1300–7708 nts; coverage: 17–558 X) from both grapevines shared 68% to 83% sequence identity with the RdRp (RNA 1; large segment), putative MP (RNA 2; medium segment) and NP (RNA 3; small segment) of ARWV2. These results suggested that one or two novel viruses related to ARWV2 were infecting the Garan dmak and Muscat rose plants. Using the *de novo* assembled contigs as starting information, preliminary genomes (one contig representing each genomic segment) of the two potential new viruses were generated and terminal ends were fulfilled by Sanger sequencing of cDNA fragments obtained using specific primers and 5′ and 3′ RACE. Once obtained the full sequences of RNAs present in both viruses, these were compared against ortholog segments of ARWV2, revealing nt identities between 56% and 77% (Table S3). Moreover, RNAs 1, 2, and 3 of the putative viruses isolated from the two grapevine cultivars shared an identity of 60%, 60%, and 57%, respectively, based on a pairwise comparison; which indicates that the viruses from the two grapevines were actually two new viruses tentatively named grapevine Garan dmak virus (GGDV) and grapevine Muscat rose virus (GMRV).

### 3.2. Genomic Organization of GMRV and GGDV

**Figure 1:**
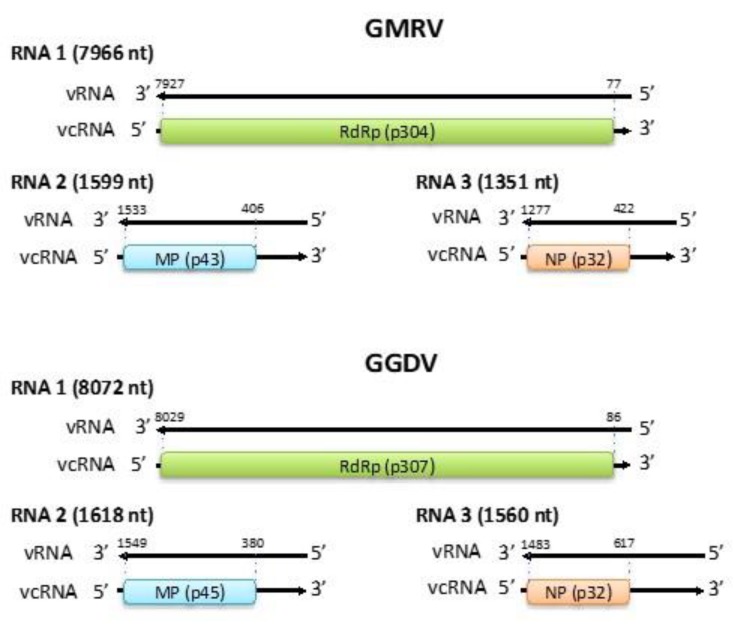
Genome organization of grapevine Muscat rose virus (GMRV) and grapevine Garan dmak virus (GGDV). vRNA, viral RNA; vcRNA, viral complementary RNA; RdRp, RNA-dependent RNA polymerase; MP, movement protein; NP, nucleocapsid protein. Nucleotide positions with respect to the vRNA are reported.

**Figure 2:**
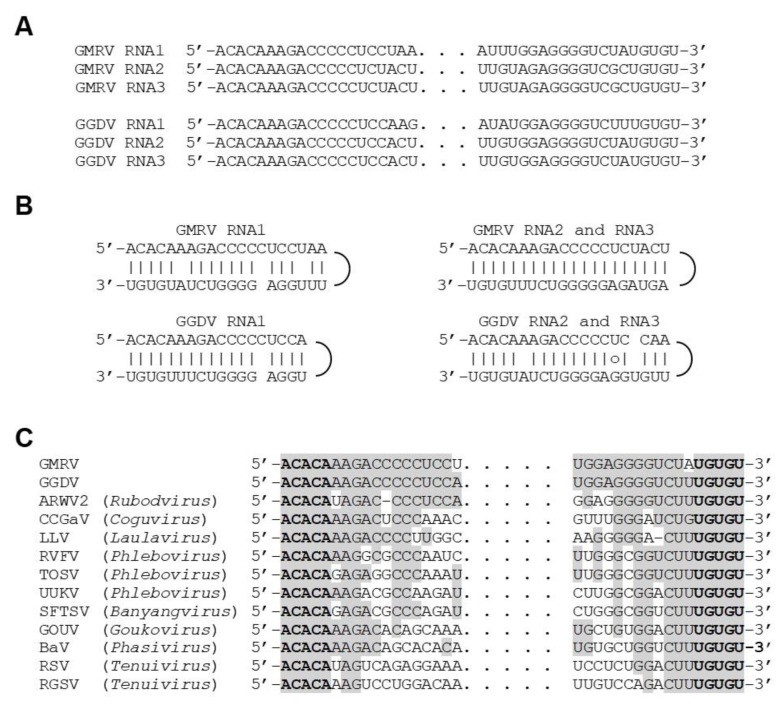
Terminal sequences of grapevine Muscat rose virus (GMRV) and grapevine Garan dmak virus (GGDV). (**A**) Alignment of 5′ (left) and 3′ (right) termini of GMRV and GGDV RNAs. (**B**) Prediction of panhandle structures formed by the 5′ and 3′ termini of GMRV and GGDV RNAs. (**C**) Alignment of GMRV and GGDV RNA 1 termini with those of other negative-stranded RNA viruses. Identical nucleotides are in grey. ARWV2, apple rubbery wood virus 2; BaV, Badu phasivirus; CCGaV, citrus concave gum-associated virus; GOUV, Gouleako virus; LLV, Laurel Lake virus; RGSV, rice grassy stunt virus; RSV, rice stripe virus; RVFV, Rift Valley fever virus; SFTSV, severe fever with thrombocytopenia syndrome; TOSV, Toscana virus; UUKV, Uukuniemi virus.

The genome of GMRV and GGDV (Figure 1) is composed by three RNA segments (RNA 1, RNA 2, and RNA 3): 7966, 1599, and 1351 nts in the case of GMRV, and 8072, 1618, and 1560 nts in the case of GGDV. The three RNA segments share almost identical nt sequences (up to 18 nt) at their 5′ and 3′ ends (Figure 2A), which are also complementary to each other and may form the panhandle structures typical of members of the order *Bunyavirales* [32] (Figure 2B). The GMRV and GGDV 5′ and 3′ genomic ends are almost identical to those reported in ARWV2, and the last five nucleotides in both termini are identical to those of other members in the family *Phenuiviridae* and the genus *Coguvirus* (Figure 2C). All three genomic RNAs of both GMRV and GGDV contain 5′ and 3′ untranslated regions (UTRs) flanking a single ORF, which based on comparisons with other nsRNA viruses is predicted to be contained in the viral complementary (vc) strand. Interestingly, the 3′ UTRs of vcRNA 2 and vcRNA 3 of both viruses are rich in A and U (65% to 67%) and particularly long (380 to 617 nts) (Figure 1), and share high sequence identity (80%) with each other, an unusual property within nsRNA viruses also shared with the RNA 2 and RNA 3 of ARWV1 and ARWV2.

**Figure 3:**
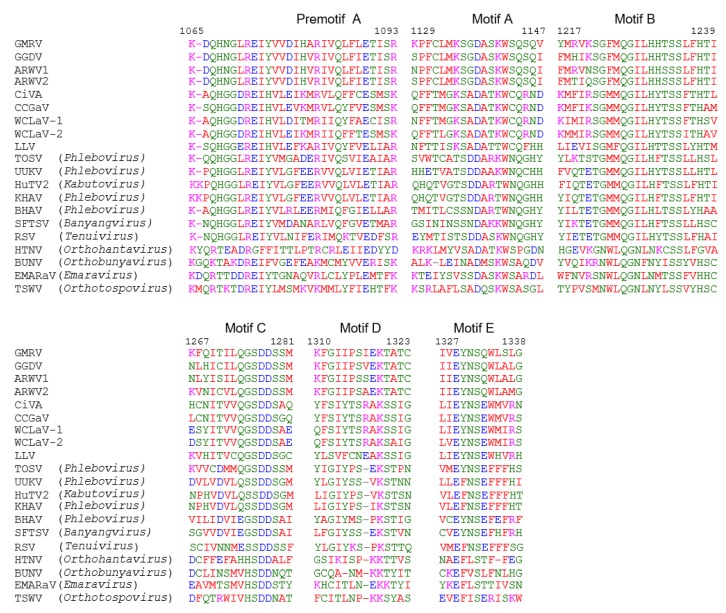
Multiple alignments of RNA-dependent RNA polymerase (RdRp) conserved motifs present in grapevine Muscat rose virus (GMRV), grapevine Garan dmak virus (GGDV) and other negative-stranded RNA viruses. Amino acid positions in the GMRV RdRp are reported. ARWV1, apple rubbery wood virus 1; ARWV2, apple rubbery wood virus 2; BHAV, Bhanja virus; BUNV, Bunyamwera virus; CCGaV, citrus concave gum-associated virus; CiVA, citrus virus A; EMARaV, European mountain ash ringspot-associated virus; HTNV, Hantaan virus; HuTV2, Huangpi tick virus 2; KHAV, Khasan virus; LLV, Laurel Lake virus; RSV, rice stripe virus; SFTSV, severe fever with thrombocytopenia syndrome; TOSV, Toscana virus; TSWV, tomato spotted wilt virus; UUKV, Uukuniemi virus; WCLaV-1, watermelon crinkle leaf-associated virus 1; WCLaV-2, watermelon crinkle leaf-associated virus 2. GMRV, GGDV, ARWV1, ARWV2, CCGaV, CiVA, RSV, TSWV, WCLaV-1, and WCLaV-2 are plant-infecting viruses.

The RNA 1 of both GMRV and GGDV encoded a putative protein of 2616 aa (304 kDa; p304) and 2647 aa (307 kDa; p307), respectively. BLASTp analysis of GMRV p304 and GGDV p307 identified the RdRp of ARWV2 and ARWV1 as the most similar proteins, with pairwise identities ranging from 54% to 67% (Table 1), a value quite below the threshold of less than 90% used as a species demarcation criteria for many genera in the order *Bunyavirales*. Pfam analysis identified the RdRp conserved domain of members of the order *Bunyavirales* (Bunya-RdRp; E-value: 3.3e-41 and 7.7e-36, for GMRV and GGDV, respectively) in both proteins. Moreover, aa alignments shown in Figure 3, revealed the presence in these proteins of the typical six motifs (premotif A and motifs A–E) highly conserved in the RdRp of members in the order *Bunyavirales*, with the highest similarity being shared with ARWV2 and ARWV1. The N-terminal region of GMRV p304 and GGDV p307 contained also the endonuclease conserved motif involved in the cap-snatching (H_303_D_303_PD_311-312_ExA_321-323_K_340_ in GMRV p304 and H_317_D_331_PD_339-340_ExT_349-351_K_368_ in GGDV p307), a strategy used by many negative-stranded viruses to translate viral proteins by using capped terminal ends from host mRNAs [33]. GGDV p307 has the ExT domain conserved in RdRp of most bunyaviruses [33], whereas in GMRV p304 this domain is replaced by ExA. Altogether, these data indicate that the putative proteins encoded by ORF 1 of GMRV and GGDV are the viral RNA-dependent RNA polymerases.

The RNA 2 of GMRV and GGDV encoded a putative protein of 375 aa and 389 aa, with a molecular mass of 43.5 kDa (p43) and 44.9 kDa (p45), respectively. The most similar proteins to p43 and p45 identified by BLASTp analysis were the putative MP of ARWV2 and ARWV1, with an aa pairwise identity between 57% to 67% (Table 1). In fact, Pfam analysis identified p43 and p45 as viral MPs (PF01107; E-value: 2.2e-13 for GMRV p43; E-value: 3.8e-13 for GGDV p45). PROMALS3D alignment of 30K MP conservative core of GMRV p43, GGDV p45, and of representative virus of several genera revealed the presence of the typical signatures of the 30K superfamily of MPs (Figure S1), consisting of an a-helix followed by seven consecutive b strands and the nearly invariant aspartic acid residue (the D motif). In addition, both proteins also contained the SIS motif near their C-termini as reported for most viral MPs included in the 30K superfamily [34]. Consequently, these data suggest the involvement of p43 and p45 in the movement within the host plant of the respective virus.

The RNA 3 of GMRV and GGDV contained an ORF coding for putative proteins of 284 and 288 aa, respectively, in both cases with a molecular mass of 32 kDa (p32). BLASTp search identified NPs of ARWV1 and ARWV2 as the proteins with the highest identity with p32. The pairwise identity between them ranged between 68% to 76% (Table 1). Accordingly, Pfam analysis identified in p32 a region significantly related to a conserved domain in the nucleocapsids of tenuiviruses and phleboviruses (*Tenuivirus*/*Phlebovirus* nucleocapsid protein; Tenui-N E-value: 5.6e-18 for GMRV, 1.2e-19 for GGDV). In agreement with this finding, when tested by Phyre2 [24], the NP of Rift Valley fever virus (RVFV; genus *Phlebovirus*) was identified as the best template for modeling (with 100% confidence) the tertiary structure of the putative NP of both GGDV and GRMV, in a region of 179 and 215 aa residues covering about 62% and 76% of the full-length protein, respectively (Figure S2). Moreover, PROMALS3D multiple alignments of p32 of GMRV and GGDV with the NP of ARWV1, ARW2 and representative members of the family *Phenuiviridae* and the genus *Coguvirus*, allowed to identify in the p32 proteins several domains/motifs reported previously to be essential for the encapsidation or infectivity of other related viruses (Figure S3). These domains/motifs included two a-helices implicated in NP oligomerization in RVFV, Uukuniemi virus (UUKV) and RSV [35,36,37], a tyrosine identified as an amino acid stacked with the 5′ end of a viral RNA in RVFV and UUKV [38,39], a phenylalanine residue shown to play an essential role in RVFV infectivity [38] and two of the three positively charged amino acids suggested to be implicated in viral RNA-binding in RVFV and RSV [37]. Notably, several of these motifs were also found in the NP of the related viruses ARWV1 and ARWV2.

### 3.3. Phylogenetic Relationships of GMRV and GGDV with Other nsRNA Viruses

**Figure 4:**
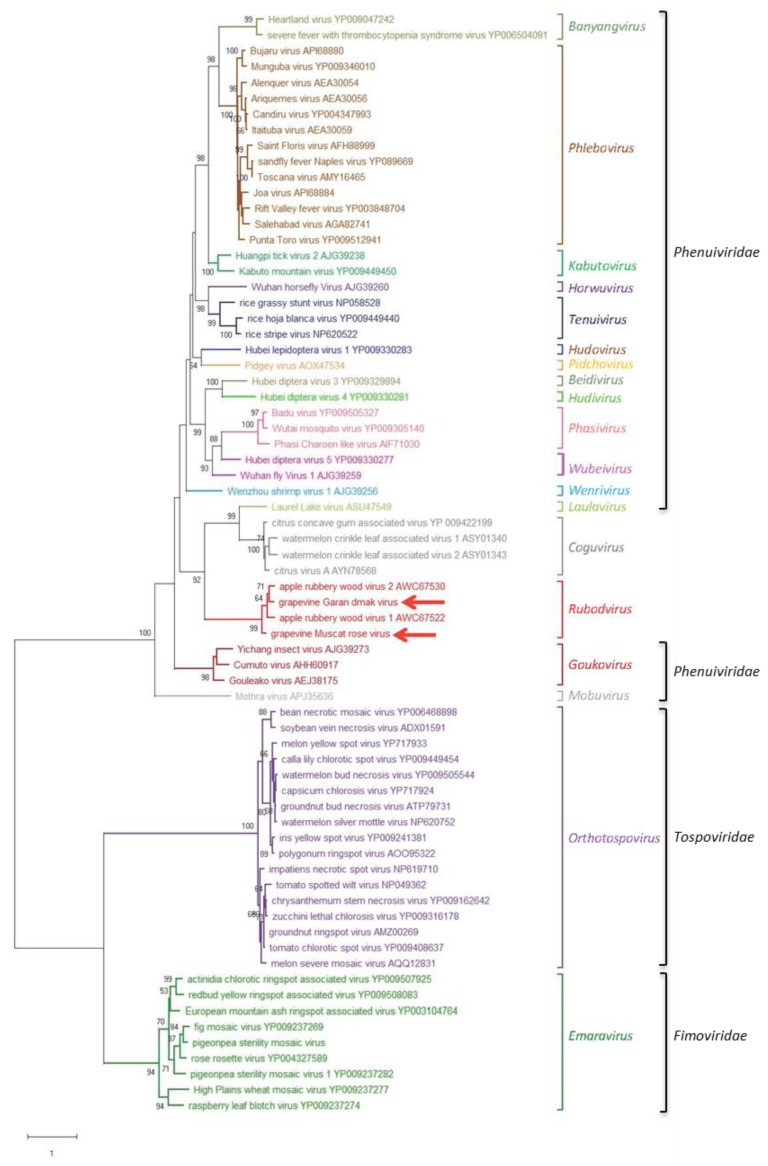
Maximum likelihood phylogenetic tree inferred from the RNA-dependent RNA polymerase conserved core domain of grapevine Muscat rose virus, grapevine Garan dmak virus and representative members of all the genera in the family *Phenuiviridae*, the plant-infecting negative-stranded RNA viruses in the genera *Emaravirus*, *Orthotospovirus*, *Coguvirus*, and the tentative genus *Rubodvirus*. The names of the viruses and the accession numbers are shown at the branch tip. Recognized and tentative genera are reported on the right, likewise, corresponding families. Bootstrap probabilities for each branch node were estimated using 500 replicates and those above 50% are shown. Tree branches are proportional to the genetic distances, with the scale bar indicating substitutions per amino acid site.

**Figure 5:**
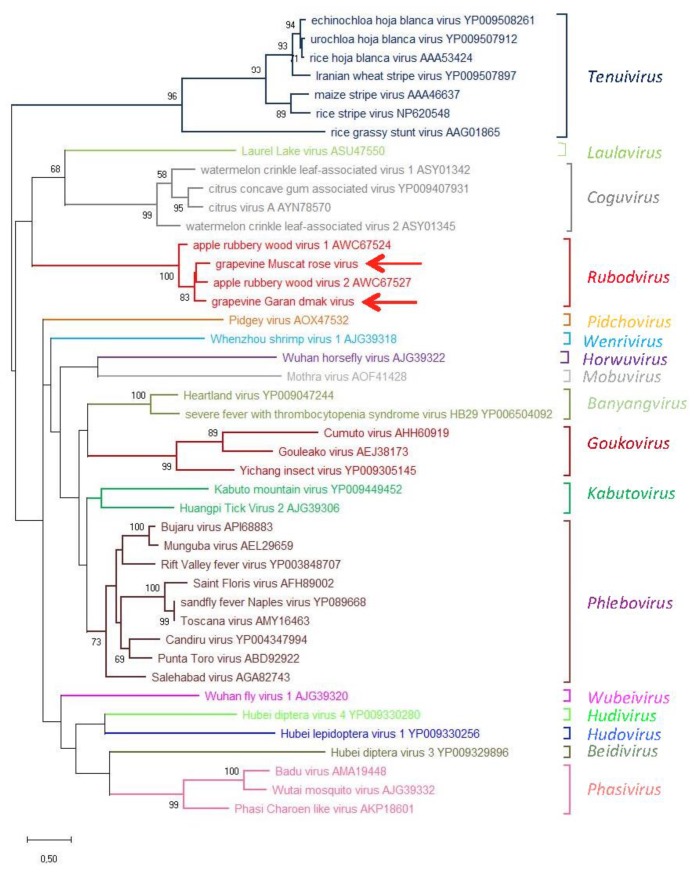
Maximum likelihood phylogenetic tree inferred from the nucleocapsid protein sequences of grapevine Muscat rose virus, grapevine Garan dmak virus, representative members of the family *Phenuiviridae*, members of the genus *Coguvirus*, and members of the tentative genus Rubodvirus. Information of bootstrap values, distances, and other symbols are reported in the legend of Figure 4.

**Figure 6:**
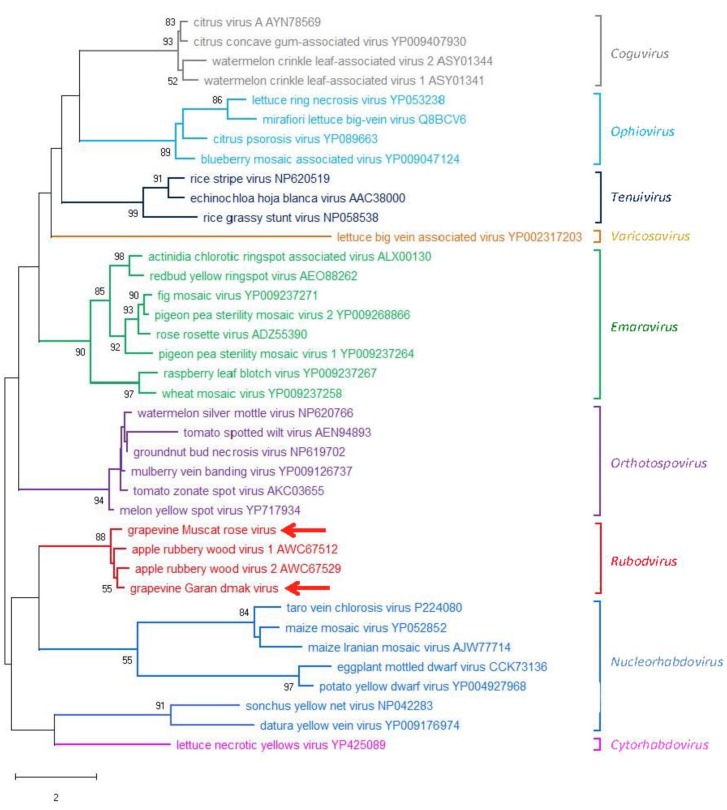
Maximum likelihood phylogenetic tree inferred from the movement protein sequences of grapevine Muscat rose virus, grapevine Garan dmak virus, and of representative plant-infecting viruses of the genera *Coguvirus*, *Emaravirus* (family *Fimoviridae*), *Ophiovirus* (family *Aspiviridae*), *Tenuivirus* (family *Phenuiviridae*), *Orthotospovirus* (family *Tospoviridae)*, *Nucleorhabdovirus, Cytorhabdovirus*, *Varicosavirus* (family *Rhabdoviridae*), and the tentative genus Rubodvirus. Information of bootstrap values, distances, and other symbols are reported in the legend of Figure 4.

An ML phylogenetic tree was inferred using the RdRp core sequence of the two novel viruses from grapevine reported here, the two viruses recently identified in apple (ARWV1 and ARWV2), representative members of all the genera in the family *Phenuiviridae* and the plant infecting nsRNA viruses in the genera *Emaravirus*, *Orthotospovirus,* and *Coguvirus* (Figure 4). In this tree, GMRV, GGDV, and the rubodviruses ARWV1 and ARWV2 form a monophyletic group with high bootstrap support (99%). In addition, this tentative rubodvirus clade is contained in a superclade that also contains coguvirus and laulavirus clades. The coguvirus clade consists of two members (citrus concave gum-associated virus and citrus virus A) and two tentative members (watermelon crinkle leaf-associated virus-1 and -2) of the genus *Coguvirus* infecting plants. The trivial (single taxon) laulavirus clade contains the arthropod-associated Laurel Lake virus (LLV; genus *Laulavirus*). The same superclade was observed when the phylogenetic tree was generated from the putative NPs encoded by GGDV, GMRV, and different recognized/tentative members of the family *Phenuiviridae* (Figure 5); with GGDV, GMRV, ARWV1 and ARWV2 as a monophyletic group, and the same phylogenetic relationship between the rubodviruses, coguviruses, and LLV. Interestingly, in the ML tree inferred using the MPs of diverse nsRNA viruses (Figure 6), rubodviruses and coguviruses are clustered in two distantly related clades.

Altogether these findings, on the one hand, highlighted the closer phylogenetic relationships between the two viruses infecting grapevine and those previously reported from apple. On the other hand, they also highlighted the different phylogenetic origin of the gene coding for MP with respect to those coding for RdRp and NP of these viruses.

### 3.4. Detection and Prevalence of Novel nsRNA Viruses

To investigate the prevalence of novel nsRNA viruses in several grapevine populations located in California, USA, different RT-PCR assays for GGDV and GMRV were developed (Table S1). Thus, specific primers that target two different regions (RdRp and NP) in the GMRV and GGDV genomes were used to screen grapevines from the NCGR, the DVC, and the FPS pipeline.

All the samples collected at the DVC and FPS pipeline tested negative. In contrast, during the survey at the NCGR, two domestic selections of Alloued zeine and Dizmar were found infected by GGDV, while a Chenin blanc grapevine originated from Argentina and infected by GMRV was identified. The positive infection status of these plants was determined by the RT-PCR-based assays mentioned above (Figure S4) and then confirmed by Sanger sequencing. Lastly, the three plants infected by GGDV and GMRV did not show any obvious symptoms. This data indicates that the two novel viruses may infect other grapevine cultivars.

### 3.5. Graft-Transmission of GMRV and GGDV

In a one-year graft-transmission test, GMRV and GGDV were successfully transmitted from the inoculum sources (Muscat rose and Garan dmak grapevines) onto four and one Cabernet franc indicator plants, respectively. These results were determined by RT-PCR using the specific primers that amplify the conserved regions in the GMRV and GGDV genomes; later, direct sequencing of PCR products confirmed the presence of the novel viruses in the grafted plants. Contrasting, non-grafted Cabernet franc plants tested negative for both viruses. No symptoms were observed on the grafted indicator plants, except for the plant that tested positive for GGDV, which developed mottle on leaves (later discussed).

## 4. Discussion

In the last few years, as a consequence of the increasing application of HTS, many novel nsRNA viruses have been identified, most of which are from invertebrates [40,41]. Thus, the classification of these viruses has been recently reassessed [42,43], with plant-infecting viruses now classified in the order *Bunyavirales* (families *Fimoviridae, Phenuiviridae*, *Tospoviridae*, and the unassigned genus *Coguvirus*), *Serpentovirales* (family *Aspiviridae,* genus *Ophiovirus*) and *Mononegavirales* (family *Rhabdoviridae*). Recently, the tentative genus Rubodvirus has been officially proposed (https://talk.ictvonline.org/files/proposals/animal_dsrna_and_ssrna-_viruses/m/animal_rna_minus_newly_submitted/8485) to classify two novel nsRNA viruses from apple trees (ARWV1 and ARWV2) [12], with a suggested species demarcation criteria of <95% aa identity for the RdRp. In the present study, HTS allowed the identification of two novel plant-infecting viruses with segmented nsRNA genome, GMRV, and GGDV, which are also the first nsRNA viruses identified and transmitted in grapevine. Although these viruses infect the same host species, the aa sequence identity between the putative proteins encoded by their genomic RNAs is always below 75% (Table 1), indicating that GMRV and GGDV are two different viruses. Their genomes encode proteins showing the highest sequence identity with ARWV1 and ARWV2. GGDV and GMRV also share other traits with these viruses, including (i) the number of genomic components, limited to three nsRNAs, (ii) identical terminal nucleotides (up to 18 nt) in the genomic RNAs, and (iii) the lack of any ORF coding for glycoproteins. Moreover, close phylogenetic relationships between these four viruses are supported by the ML phylogenetic trees reported here, in which, independently of the considered protein (RdRp, NP, or MP), they always clustered in the same clade, which is significantly separated from all the other nsRNA viruses included in the analyses. Altogether these data support the classification of GMRV and GGDV as two novel species in the tentative genus Rubodvirus.

When the other bunyavirales are considered, the ML phylograms inferred from the RdRps or the NPs (Figure 4 and Figure 5) show a close phylogenetic relationship between rubodviruses and coguviruses, which together with the arthropod-infecting LLV, form a superclade nested at a basal node, in closer proximity to arthropod-infecting viruses than other plant-infecting viruses. These data are consistent with the hypothesis, previously advanced for the coguviruses [9,10], that all the members of this superclade evolved from a common ancestor virus infecting arthropods. In this evolutionary scenario, the acquisition of the MP gene appears to be the key step in the adaptation of the ancestor virus to plants [44,45], an event that likely happened through the typical modular genome evolution process proposed for most eukaryotic viruses [45]. However, the clustering of rubodviruses and coguviruses in two distant clades, observed in the ML tree inferred with MPs (Figure 6), supports the independent acquisition of the MP gene by the ancestor of the viruses included in these two taxa. These data are consistent with the hypothesis that the adaptation of invertebrate-infecting nsRNA ancestor viruses to plants happened several times through independent events during the evolutionary history of nsRNA viruses infecting plants [9,10].

It is worthy of note that rubodviruses, laulaviruses, and coguviruses, although phylogenetically related, have divergent genome structures and gene expression strategies. In fact, the members of the first two genera have a genome composed of three monocistronic nsRNAs encoding different proteins; RdRp, NP, and putative MP in the case of rubodviruses, and RdRp, NP and a protein of unknown function in the case of laulaviruses [46]. Instead, coguviruses have a bipartite genome consisting of one nsRNA encoding the RdRp (RNA 1), and one ambisense RNA (RNA 2), in which the ORFs encoding the NP and the MP are separated by a long intergenic region (IR). It has been shown that such an IR is AU-rich and self-complementary, thus assuming in both polarity strands a compact conformation containing a long hairpin predicted to serve as a transcription termination signal during the expression of viral genes [9,10]. Taking this into consideration, the question arises of how viruses with such different genomic organizations may have evolved from the same ancestor virus. In this respect, it can be speculated that a recombination event between the viral and the vc strand of two genomic RNAs with long, AU rich, and almost identical 5′ UTRs could generate ambisense RNAs containing an IR similar to those of coguviruses. Since nsRNA viruses with genomic RNAs showing structural features (long and almost identical 5′ UTRs) compatible with this possibility were not known previously, such a possibility appeared unlikely. However, the very long, AU-rich, and highly conserved 5′ UTRs reported here for RNA 2 and RNA 3 of GMRV and GGDV, and also observed in the corresponding RNAs of ARWV1 and ARWV2, are the first clear evidence that nsRNA viruses with the structural features compatible with this evolutionary scenario may exist. Based on these considerations, the possibility that the bipartite genomes of coguviruses originated from a tripartite ancestor with genomic RNAs containing 5′ UTRs similar to those observed in the rubodviruses appears feasible.

No glycoprotein is encoded by rubodviruses (ARWV1, ARWV2, GMRV, and GGDV), a feature previously reported also for coguviruses that, according to electron microscopy observations, are flexuous, non-enveloped viruses [9]. In contrast, glycoproteins are expressed by most nsRNA plant viruses transmitted by arthropods (nucleorhabdoviruses, cytorhabdoviruses, emaraviruses, tospoviruses, and tenuiviruses). The lack of glycoprotein in the genome of rubodviruses and coguviruses opens the question on the existence of vectors, if any, involved in their transmission. In this respect, it is worthy of note that ophioviruses and varicosaviruses, which are plant-restricted or transmitted by fungi [47], also do not code for any glycoprotein. Vegetative propagation has been proposed as the prevalent transmission mechanism for ARWV1 and ARWV2 [12]. Whether, this is also the case for GMRV and GGDV needs further investigation.

Most plant viruses code for viral suppressor proteins (VSRs) counteracting the plant antiviral defense mechanisms based on RNA silencing [48]. Further studies are needed to ascertain whether one or more of the three proteins encoded by GGDV and GMRV and other rubodviruses may interfere with RNA silencing, thus showing multifunctional role(s), as already reported for VSRs of other viruses [48].

GMRV and GGDV were identified in two different grapevines cultivars, Muscat rose and Garan dmak, that were tested by HTS; moreover, both viruses were found in association with other viruses and viroids. Interestingly, no obvious symptoms were observed in the two grapevines. Although infectivity of both viruses was ascertained by graft-transmission, only a Cabernet franc grapevine infected by GGDV developed symptoms (leaf mottle) post-grafting; further HTS analysis on this indicator plant reveals the presence of GRSPaV, GYSVd-1, and HSVd. Therefore, it was not possible to ascertain whether GGDV is associated with symptoms and additional studies on its pathogenicity are needed, likewise GMRV.

An initial survey using several accessions of grapevine located in three different collections in California resulted in the identification of two and one plants infected by GGDV and GMRV, respectively; like the original sources of the viruses (Muscat rose and Garan dmak selections), these grapevines were symptomless. A more extensive survey, including other grapevine-growing regions in California and the USA, is necessary to determine the real distribution of these novel viruses. In that sense, the detection method (RT-PCR) developed in this study could be useful for virus testing and certification programs. Finally, during the review process of this manuscript, two novel mycoviruses related to coguviruses and rubodviruses were reported [49,50], which extends the host range of phenui-like viruses.

## Figures and Tables

**Table 1:** Amino acid sequences identities between grapevine Muscat rose virus, grapevine Garan dmak virus, and apple rubbery wood viruses 1 and 2.

Virus *		BLASTp Results									Pairwise Identity (%)			
		RdRp			MP			NP			RdRp	RdRp Core	MP	NP
		E Value	Identities		E Value	Identities		E value	Identities					
GGDV	Apple rubbery wood virus 2	0.0	1610/2384 (68%)		0.0	320/389 (82%)		5.00E-175	258/288 (90%)		67.24	78.57	67.10	75.87
	Apple rubbery wood virus 1	0.0	1383/2380 (58%)		8.00E-162	241/385 (63%)		5.00E-139	196/284 (69%)		58.00	74.39	60.68	68.18
	Grapevine Muscat rose virus	0.0	1411/2660 (53%)		2.00E-172	226/387 (58%)		6.00E-156	211/284 (74%)		53.48	70.41	59.52	74.30
														
GMRV	Apple rubbery wood virus 2	0.0	1337/2373 (56%)		6.00E-166	228/375 (61%)		6.00E-142	210/284 (74%)		55.83	69.94	59.20	73.94
	Apple rubbery wood virus 1	0.0	1306/2387 (55%)		8.00E-155	226/390 (58%)		3.00E-142	194/284 (68%)		54.60	70.61	57.33	67.96
	Grapevine Garan dmak virus	0.0	1411/2660 (53%)		2.00E-172	226/387 (58%)		6.00E-156	211/284 (74%)		53.48	70.41	59.52	74.30

GMRV, grapevine Muscat rose virus; GGDV, grapevine Garan dmak virus; RdRp, RNA-dependent RNA polymerase; MP, movement protein; NP, nucleocapsid protein. RdRp core corresponds to the region containing the typical signatures of the RdRp present in members of the order *Bunyavirales* and obtained by Pfam analysis (Pfam family: Bunya-RdRp; PF04196). * Apple rubbery wood virus 2 (RdRp: AWC67514.1, MP: AWC67531.1, NP: AWC67526.1); apple rubbery wood virus 1 (RdRp: AWC67516.1, MP: AWC67523.1, NP: AWC67524.1).

## Supplementary Materials

The following are available online at https://www.mdpi.com/1999-4915/11/8/685/s1, Figure S1: Multiple sequence alignment of putative MPs encoded by GMRV, GGDV, and 30K MPs encoded by viruses representative of several genera, Figure S2: Modeling and prediction of NPs encoded by GGDV and GMRV, Figure S3: Multiple sequence alignment of NPs encoded by GMRV, GGDV, and NPs encoded by viruses representative of several genera, Figure S4: Detection of GMRV and GGDV in grapevine plants by RT-PCR, Table S1: PCR primers used for the detection of novel nsRNA viruses in grapevine, Table S2: HTS data generated from the Muscat rose and Garan dmak grapevines, Table S3: Nucleotide sequences identities between GMRV, GGDV, and ARWV2.
