# Supplementary material for: Two Novel Negative-Sense RNA Viruses Infecting Grapevine Are Members of a Newly Proposed Genus within the Family Phenuiviridae
Source: Viruses. 2019 Jul 26;11(8):685. doi: 10.3390/v11080685 (PMC6724010; doi:10.3390/v11080685)
Supplement: Supplementary file 1 [file viruses-11-00685-s001.zip › Figure S1.pdf]

```
 ID     GenBank       Genus      aa position
GMRV                             113 ------YRPI---------DVNERL---KMS-------------AKYRTVHLDGIMVNCESLMPK-----
GGDV                             115 -----VYHPI---------DSNERI---RMR-------------KHYRTVHLDGIEVNCESLMPK-----
CaLCuV NP_624353    Begomovirus     38 ----LTEILLQ------FPSTAAQF--TARL--------------NRSCMKIDHCVIEYRQQVPI-----
CiLV-C YP_654542    Cilevirus       47 ---RIGVGFLT------PNDMISRL--VGFI---NRKAEDAGVRSVESFRQISDVVLIIVPQIA------
AcVB   YP_004935360 Vitivirus       78 ---VDQNIDLV------DEKTIDGL----------NEE-------KQPYLHLGCVAIAVIPHGR------
LNYV   YP_425089    Cytorhabdovirus 53 ---IMRQVPLF------DKEDLDAM--KS------DTT-------SNKYLHIGCITVSIEPLLHQRYMKN
AMV    NP_041194    Alfamovirus     60 ---APVVLSLV------SKEKKSIL--NRML---PKIG-------QRMYVHHSAIYLLYMPNILK-----
CMV    NP_040776    Cucumovirus     52 ---AISVRPLV------PEVTHGRI--ASFF---KSGYDVGE-LCSKGYMSVPQVLCAVTRTVST-----
CaMV   NP_056724    Caulimovirus    97 ---GKVYLPLI------TKEEINKR--LSSL---KPEVR-----KTMSMVHLGAVKILLKAQFRN-----
ToTV   YP_001040018 Torradovirus    66 ----KKAMELF------DPEDVHNI--TSLWSSFKKTFTSSR-DHGNLFFHLYGVMFFMVPHVHG-----
TBSV   NP_062900    Tombusvirus     32 ---EDQEVPLL------PSNFLTKV---RVG-------------LSGGYITMRRIRIKIIPLVSR-----
RBDV   NP_620466    Idaeovirus     138 ---GKNEFSLV------EASVFDKL--IR---------------KDDSPIHLNRLLIAVLPAVGK-----
PYFV   NP_734443    Sequivirus     115 ----RAEITPT------AASKLDKT--LE------AHRA------KFNYMAIDSIRVAVTSLMHQ-----
CLBV   NP_624334    Citrivirus      55 ---IKLQANIL------TADRLQSI--RN------AKVNGK----EAAYLHLGFVPIAIRSLLPS-----
CCGaV  YP_009407930 Coguvirus      122 DDTEQFNLSLA------VPKKG-----------------------KQYVRLSSVMAYYCPLVSS-----
TMV    NP_597748    Tobamovirus     36 -CSKVDKIMVHENESLSEVNLLKGV--KL---------------IDSGYC-LAGLVVTGEWNLPD-----
RRV    YP_004327592 Emaravirus      63 ---KTQLLPIS------VYNEMYKF--YM------HLK--------QPMTRIASVALFWTPTSK------
PYMoV  YP_008567619 Badnavirus     100 ---NQVDRTFI------QEESHKKL-----------VKS------GIKFIHLGVLQVRLQILHRK-----
SDV    NP_733976    Sadwavirus      53 ---QQQDIAIP------LSSRFG---------------------NHQSLHVGELEIAVQSSVLT-----
ACLSV  NP_040552    Tricovirus      62 ---RLASIPVI------PSSEVQAV---------LRKRE------STNYVHWGALSISIDALFRK-----
RSV    NP_620519    Tenuivirus      88 -----ATFNIL------VDPYWFHQPLTHYPF-FRVTHYP-----FFRVETFAMVWIGIKG---------
GFLV   NP_733995    Nepovirus A    118 -NTLEILYNPV------SEEEMEEY--RDR---------------GMSAVVIDALEIAINPFGMP-----
CRLV   YP_081449    Cheravirus     109 -DPYVAVYTPLP---HITEEQARKL--MEK---------------GFSNSSNVALDMAVQSHVGQ-----
CPMV   NP_734000    Comovirus      204 ---TVLDIPVT------KDKTLAMA--SD-----FIRKENL----KTSAIHIGAIEIIIQSFASP-----
CPsV   YP_089663    Ophiovirus      84 ---HKKKLKLG------TLKSITDK--LR-----KLGGES-----SQPFIQFYKVQCMYIPLFSRV----
PoLV   YP_009032637 Aureovirus      30 ---TKAILPLA------PISQFSKW----------KIP-------KQGFYAPIDVKFVLTPHISE-----
OuMV   YP_002019758 Ourmiavirus     57 ---VEDPIALI------PHGIWSIF--KSKL---AQMRC------PKGYITYDKVILSWKPHVAT-----
CNSV   NP_733974    Nepovirus B    432 ------VLNRP------EYNALNKL--A------EAGWK---------EAKSVVLNLHIRSYLPQ-----
CMoV   YP_002302261 Umbravirus      42 ---TLTHTPLY------PPRRQLTL--SRFF---SGRWRTK---KTGGMLFIEKLVIVFVPHVPD-----
SBWMV  NP_049337    Furovirus       44 ---RYVEKALT------QPGVVTKM--QDAW---TNWTKTNKEEGTPYNMSYSCVLLNVIPTVPM-----
RCNMV  NP_620546    Dianthovirus    33 ---GVSEAPLI------PASMMSKI--TDYA---KTTAK-----GNSVALNYTHVVLSLAPTIGV-----
TSWV   AEK06236     Orthotospovirus 111 ---ANKVIKIC-----------------------PTWD-----SRKQYMMISRIVIWVCPTIPN-----
Consensus ss:                                 ee          hhhhhhh   h                eeeeeeeeeeee
```

```
GMRV                                 -GSEGFAIMTLYDERFISQ------EKGFLG--LIGFPLSHGV-------SKASLKIN------------
GGDV                                 -GSEGFAILTLYDERFVSQ------EKGFLG--LIGFPLSHGV-------SKASLKVN------------
CaLCuV NP_624353    Begomovirus      -NATGSVIVEIHDKRMTD-------DESLQA--SWTFPLRCNI-------DLHYFSSS------------
CiLV-C YP_654542    Cilevirus        --LPAELSLKLVDSANI--------LEAVNQ--EVTVNSTGGP-------CVVVMNCA------------
AcVB   YP_004935360 Vitivirus        -AMKGTVQIKVEDQRFKE-------GHGTVC--SFKCDLKDAL-------SAYASFPG------------
LNYV   YP_425089    Cytorhabdovirus FGKTIAGNCAIIDSTFRKV------DQSIIS--LHKYDLSRGR-------ADYVSYPN------------
AMV    NP_041194    Alfamovirus      -SSSGSITLKLFNEATGEL---------VDV--DTDHDATQA--------CIFAGRYP------------
CMV    NP_040776    Cucumovirus      -DAEGSLRIYLADLGDKEL------SPIDGQ--CVSLHNHDLP-------ALVSFQPT------------
CaMV   NP_056724    Caulimovirus     -GIDTPIKIALIDDRINSR------RDCLLG--AAKGNLAYGK-------FMFTVYPK------------
ToTV   YP_001040018 Torradovirus     --GEGSVKISLCSSNDP--------TNPVLQ--EKVLYFSGGA-------QAVLMSPT------------
TBSV   NP_062900    Tombusvirus      -KAGVSGKLYLRDISDTT-------GRKLHC--TESLDLGREI-------RLTMQHLD------------
RBDV   NP_620466    Idaeovirus       -GTPGTARIKIRDARLDDG------YGELFS---SENRVDSG--------VIYCINVG------------
PYFV   NP_734443    Sequivirus       -GDSRECIMYLCDRRFKDP------LLGAIA--LIGFTLPGMQTHVYKTGRMMAFSRK------------
CLBV   NP_624334    Citrivirus       GNEQIWGRCALVDTSRTRA------ETAVID--EFEFKFTKKQPF---ASKLLTINAAVD---------I
CCGaV  YP_009407930 Coguvirus        FSEFTKVSISLHDSRLLS--------KTCVQ--SADFNSNIT--------QKVELSLD------------
TMV    NP_597748    Tobamovirus      -NCRGGVSVCLVDKRMERA------DEATLG--SYYTAAAKKR-------FQFKVVPN------------
RRV    YP_004327592 Emaravirus       -RFNEMATLILMDERFRDDGI-KQGKKELLA--QGKLNLDLMG-------TVITAVPFDPNFQQFVTGSL
PYMoV  YP_008567619 Badnavirus       -EEGTLALVVFRDNRWKG-------DKGIIA--TMEVDLTKGC-------QMVYIIPD------------
SDV    NP_733976    Sadwavirus       -GVDTAMAIMVSDASHDRL------EEGFLS--LTILRLGAGW-------MRHTIPIG------------
ACLSV  NP_040552    Tricovirus       -NAGVSGWCYVYDNRWETF------EQAMLQ--KFHFNLDSGS-------ATLVTSPN------------
RSV    NP_620519    Tenuivirus       -RASGITTLRIIDKSYVNP------SDQVEV--EVRYPISKNF-------AVLGSLAN------------
GFLV   NP_733995    Nepovirus A      -GNPTDLTVVATYGHERDM------TRAFIG--SASTFLGNGL-------ARAIFFPG------------
CRLV   YP_081449    Cheravirus       -GTPLLAMCGIMDSRTDDP------NEALQV--AGYFDLGRDR-------CDLISLPL------------
CPMV   NP_734000    Comovirus        -ESDLMGGFLLVDSLHTDT------ANAIRS--IFVAPMRGGR------PVRVVTFPN------------
CPsV   YP_089663    Ophiovirus       DGDNGEITVSLIDDGKEAA-----GQDPIIQ--SITFDASQM--------AMVELSMN------------
PoLV   YP_009032637 Aureovirus       -RAQVRGVVKLVDSRDLSP------SRELYR--SKEFNIGHGL-------VIEGSQLP------------
OuMV   YP_002019758 Ourmiavirus      --GLARGQIAVVDTRVNHTSIEDLMHKALWK--TAPVDLGCTY-------TIQGTVP-------------
CNSV   NP_733974    Nepovirus B      -QMNAYAFCVIMWGHSSDA------QEAALS--GSYVYLGDGE-------ATMLQLPL------------
CMoV   YP_002302261 Umbravirus       -TSPGQAVIWVHDTALPGL-------EPIGTGQKVCIPLSSGP-------RLVAFYPN------------
SBWMV  NP_049337    Furovirus        -GYAGTVEVSLLDSGLSPL-------ENVIPDQTQMMELGKGP-------NVMCFFMH------------
RCNMV  NP_620546    Dianthovirus     -AIPGHVTVELINPNVEGP-------FQVMSGQTLSWSPGAGK------PCLMIFSVH------------
TSWV   AEK06236     Orthotospovirus --PTGKLVVALVDPNMPSE-------KQVIL--KGQGTITDP--------ICFVFYLN------------
Consensus ss:                            eeeeeee                 eee   eeeee              eeeee
```

```
GMRV                                 -YSISTADN------------VNWVAIITVYDHCLRD-DMRPCNFHLKAFYKYTNNVRRFIKDEEKSEIG
GGDV                                 -YSISTFDE------------VNWVAVITVYDHNLRD-DMRPCNFHLKAHYKYTNNVSRFLKNIDETEVG
CaLCuV NP_624353    Begomovirus      -FFSLKDPI-------------PWKLYYRVSDTNVHQ-RTHFAKFKGKL---------------------
CiLV-C YP_654542    Cilevirus        -HSIPNEDRTHVNG---SEVHRRLGIQYQVDCDNISG-RVTTFSITALW---------------------
AcVB   YP_004935360 Vitivirus        -YFVSTTDVKN---------GYALNLKVKAEGMCMVE-GVHPLSIQMHC---------------------
LNYV   YP_425089    Cytorhabdovirus -HCLSLTDPMI---------QKRLSVLLGIKGIDVEP-GVELFSICIGY---------------------
AMV    NP_041194    Alfamovirus      -RSILAKDAAK---------GHDLKLVVHAVASTNA--NSAVGVLYPIW---------------------
CMV    NP_040776    Cucumovirus      -YDCPMETVGN--------RKRCFAVVIERHGYIQY--TGTTASVCSNW---------------------
CaMV   NP_056724    Caulimovirus     -FGISLNTQRL---------NQTLSLIHDFENKNLMNKGDKVMTITYVV---------------------
ToTV   YP_001040018 Torradovirus     -ITLPFVKRG-----------PMFYYTMECLGTRA----QIPCSVVAIW---------------------
TBSV   NP_062900    Tombusvirus      -FSVSTRSD------------VPIVFGFEELVSPFLE-GRELFSISVRW---------------------
RBDV   NP_620466    Idaeovirus       -YSVPKSEI-------------DYKINIDFAGVPIKD-GKSPIWVKAAF---------------------
```

```
PYFV   NP_734443    Sequivirus      -EAIAAD---------------RLQLYLYVKGAKLERTQNTPITVNVRT---------------------
CLBV   NP_624334    Citrivirus      NCKVSVG---------------SIQVLLELHGVDLRE-ERSVAAII------------------------
CCGaV  YP_009407930 Coguvirus       -YCIPRTSC------------SKITLNIAREQKFLQE-GEEWATVQLLIRL-------------------
TMV    NP_597748    Tobamovirus     -YAITTQDAMK----------NVWQVLVNIRNVKMSA-GFCPLSLEFVSVCIVY----------------
RRV    YP_004327592 Emaravirus      DFATDTKDI------------NKIKFYISFPDTRM-----------------------------------
PYMoV  YP_008567619 Badnavirus      -IMMTVNDFY-----------HNIHISVLTRGYEGWQHGEANLLVTRGM---------------------
SDV    NP_733976    Sadwavirus      -ITVFPTDPLV---------DRFLRLSVLTGGSPMAD-GRQVARLHYGL---------------------
ACLSV  NP_040552    Tricovirus      -FPVSLDDPGL---------SNSISVAVMFENLNFKF-ESYPISVRVGN---------------------
RSV    NP_620519    Tenuivirus      --FLALEDK------------HNLQVSVSVDDSSVQN---CVISRTLWFW--------------------
GFLV   NP_733995    Nepovirus A     -LQYSQEEPRR---------ESIIRLYVASTNATVDT-DSVLAAISVGT---------------------
CRLV   YP_081449    Cheravirus      -INFPLNKEDFD------DYMRGLYLCTMFHNVRGFQNNKALCSYSA-----------------------
CPMV   NP_734000    Comovirus       -TLAPVSCDL----------NNRFKLICSLPNCDIVQ-GSQVAEVSVNV---------------------
CPsV   YP_089663    Ophiovirus      -FFVEKKDM------------DFIGIHVSAENVPVQ--DRAYGSINLAFF--------------------
PoLV   YP_009032637 Aureovirus      -FCLPVGD-------------YPLQFEVTVLQSQFRE-TANLYSTSVEW---------------------
OuMV   YP_002019758 Ourmiavirus     -YCLPFHPKEGGDVKSDLESQNPIRGIVYITDSRY-----------------------------------
CNSV   NP_733974    Nepovirus B     -LCEYVGHNLQDF----EAYKRSLVLSTVFPEFSGIADGKAMFGITSIEF--------------------
CMoV   YP_002302261 Umbravirus      -YSIPLSDSAMN-------APRCFSLVTQLEGVRLEQ-GASAFSLYSMW---------------------
SBWMV  NP_049337    Furovirus       -YSIPLNDKGR---------AVKLAFKIDAEM--ASK-GMSVMNVYSYW---------------------
RCNMV  NP_620546    Dianthovirus    -HQLN--SDH-----------EPFRVRITNTG--IPT-KKSYARCHAYW---------------------
TSWV   AEK06236     Orthotospovirus -WSIPKMNNTP---------ENCCQLHLMCSQ-EYKK-GVSFGSVMYSW---------------------
Consensus ss:                         eee                  eeeeeeee         eeeeeee
```

Figure S1. Multiple sequence alignment of movement proteins (MPs) encoded by grapevine Muscat rose virus (GMRV), grapevine Garan dmak virus (GGDV) and 30K MPs encoded by viruses representative of several genera. The virus name, GenBank identifier, virus genus and distance in amino acids (aa) from the N-terminus of the protein are shown on the left-hand side of each sequence. Consensus secondary structure (ss) elements, predicted by PROMALS3D, are reported at the bottom, with the strands and helices indicated by “e” and “h”, respectively. Amino acids that fold in strands and helices and are in blue and red, respectively.
