# Supplementary material for: Two Novel Negative-Sense RNA Viruses Infecting Grapevine Are Members of a Newly Proposed Genus within the Family Phenuiviridae
Source: Viruses. 2019 Jul 26;11(8):685. doi: 10.3390/v11080685 (PMC6724010; doi:10.3390/v11080685)
Supplement: Supplementary file 1 [file viruses-11-00685-s001.zip › Figure S2.pdf]

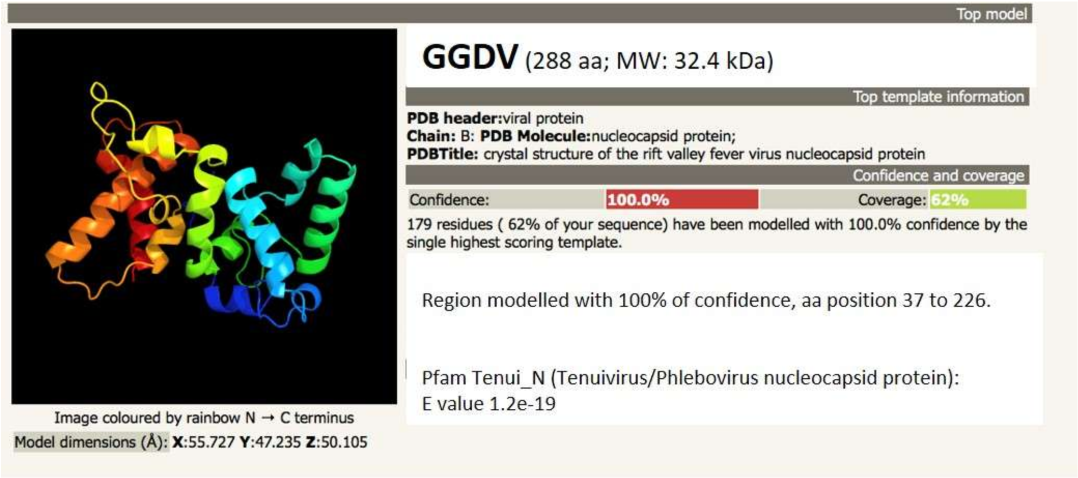

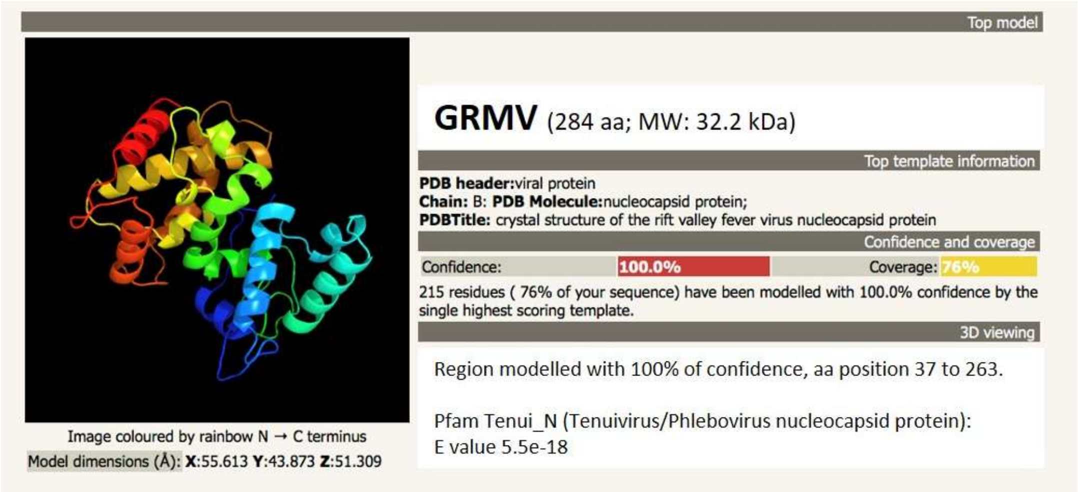

Figure S2. Modeling and prediction of nucleocapsid proteins (NPs) encoded by grapevine Garan dmak virus (GGDV) and grapevine Muscat rose virus (GMRV). Analysis performed with Phyre2 web portal.
