# Supplementary material for: Two Novel Negative-Sense RNA Viruses Infecting Grapevine Are Members of a Newly Proposed Genus within the Family Phenuiviridae
Source: Viruses. 2019 Jul 26;11(8):685. doi: 10.3390/v11080685 (PMC6724010; doi:10.3390/v11080685)
Supplement: Supplementary file 1 [file viruses-11-00685-s001.zip › Figure S3.pdf]

```
GGDV             1  MAD-----------------GNFA--LTGL----------------------------------------   11
GMRV             1  MAN-----------------GDFL--LAEF----------------------------------------   11
ARWV1            1  MAT-----------------GDFT--ISSF----------------------------------------   11
ARWV2            1  MAN-----------------GDFV--LSGL----------------------------------------   11
UUKV             1  MAM-------------------------------------------------------------------    3
SFNV             1  MSDE------------------------------------------------------------------    4
TOSV             1  MSDE------------------------------------------------------------------    4
RVFV             1  MD--------------------------------------------------------------------    2
SFTSV            1  MS--------------------------------------------------------------------    2
CiVA             1  MALHQNPKE--QSKFDALFSQATSHGEEHYREFLTKCSKNLKERIKREVNARK--MTSGGDFLESFEQAE   66
CCGaV            1  MADKINNPS-TQAEFEAIYHDATSQDQAYFVAYFKRCSKALQERLKKEISARK--LIGAG----------   57
WCLaV_1          1  MD---HTN----AELIALINEKSSLGQAEFNKWFESLSKNNKSYVQKEMKARK--LAKAMTPSA-VEATE   60
WCLaV_2          1  MSSSSRTNNTLREQMKALKNKPES----EQSAWLEGLNEQEMRFVETQMKTTKGIKLAQTTAASSSETSE   66
UHBV             1  MAM----------------------SVADVQTEIE-----------------------------------   13
IWSV             1  MSM----------------------SVAEIQAEIE-----------------------------------   13
RHBV             1  MTM----------------------SVADVQTEIE-----------------------------------   13
EHBV             1  ------------------------MSVADIQTEIE-----------------------------------   11
RSV              1  MGT----------------NKPAT--LADLQKAIN-----------------------------------   17
MSV              1  MAT----------------NKPAN--LNDLQKAIN-----------------------------------   17
RGSV             1  MGK------------------------VQFGDGHW---ANNK----------------------------   15
LLV              1  MT--------------------------------------------------------------------    2
Consensus_ss:                                   hh


GGDV            12  ------------------------------------TTALTLINHLLTVSATNFK--AAARANT------   37
GMRV            12  ------------------------------------TTVITLLSHLLDVSAANYR--SAAKKNA------   37
ARWV1           12  ------------------------------------STVVTLLNHLLTVSAANFK--AAAKQNA------   37
ARWV2           12  ------------------------------------TTALTLVNHLLTVSAENFK--SAARSNA------   37
UUKV             4  ---------------------------------PENWVRFAI--EISDAQWEE----EEIREFI------   28
SFNV             5  -----------------------------------NYRDIAL--AFLDESADS----GTINAWV------   27
TOSV             5  -----------------------------------NYRDIAL--AFLDESADS----GTINAWV------   27
RVFV             3  -----------------------------------NYQELAI--QFAAQAVDR----NEIEQWV------   25
SFTSV            3  -----------------------------------EWSRIAV--EFGEQQLNL----TELEDFA------   25
CiVA            67  DKSGKLDNKEGELEEDEHEDEEQEVTEGSGTKKDIVVTDAQL--AAMWTEIDN----IDVSTFDRE---S  127
CCGaV           58  PKFATRDG--DDGQEPENKGDEA---------DSSNISDIQL--AEMWQEIDL----MDVTSIDSE---S  107
WCLaV_1         61  ETSSS--------SRPPTQSIPKP-------VLTDEVSDSDL--ISMWSEIDSMDVSSIDRESL------  107
WCLaV_2         67  DPKG-----------PAVESLP------------TEITEARI--AAMWTEIDN----FDVSSLDQE---S  104
UHBV            14  -----------------------------------RVTALAL--KYITDHKD------TLVTFV------   34
IWSV            14  -----------------------------------RVSTLAL--KYITEHKD------ILVAFA------   34
RHBV            14  -----------------------------------RVTTLAL--KYITDHKD------TLVTFV------   34
EHBV            12  -----------------------------------RVTALAL--KYIADHKD------TLVTFV------   32
RSV             18  -----------------------------------DISKDAL--SYLTAHKA------DVVTFA------   38
MSV             18  -----------------------------------DISKDAL--KYLTDNKA------SVTTFH------   38
RGSV            16  -----------------------------------EWSDLLS--EIFSKIR------ASIDGFANATADL   42
LLV              3  -------------------------------------SALSV--YTMISAADT----TGVEEFS------   23
Consensus_ss:                                          hhhhhhh  hhhhhhhhh    hhhhhhh


                         *                                       *              *
GGDV            38  -KYFAYDGFNANEILTSFIAIFL-TNSNDDDALQDLCDIVNIGLTRGNI-----REDQLKKTS--KRGRE   98
GMRV            38  -KEFAYDGFNANEILNSFLKSFK-ANEDDEEALQDLCDIVNIGLTRGNI-----RADQLSKTS--KKGRE   98
ARWV1           38  -KYFAYDGFNAIEILTAFLIIFK-SNTDDADALQDLCDIVNIGLTRGNI-----REDQLQKTS--KKGKE   98
ARWV2           38  -KYFAYDGFNASEILTAFITIFS-GNTDSDDALQDLCDIVNIGLTRGNI-----HENQLKKTS--KRGRE   98
UUKV            29  -NLFQYQGFDAAVVLSRIFELAKKADLSRDQMLRDIRALITLHLTRGNK-----LSSIEKRLS--EEGKK   90
SFNV            28  -NEFAYQGFDPKRIVQLVKERGT---AKGRDWKKDVKMMIVLNLVRGNK-----PEAMMKKMS--EKGAS   86
TOSV            28  -NEFAYQGFDPKRIVQLVKERGT---AKGRDWKKDVKMMIVLNLVRGNK-----PEAMMKKMS--EKGAS   86
RVFV            26  -REFAYQGFDARRVIELLKQYG------GADWEKDAKKMIVLALTRGNK-----PRRMMMKMS--KEGKA   81
SFTSV           26  -RELAYEGLDPALIIKKLKET------GGDDWVKDTKFIIVFALTRGNK-----IVKASGKMS--NSGSK   81
CiVA           128  TKVFDYQGFNPDEVLRSLIINQRKNKVSAADFKSDILLMCSLAIIKGSI-----NEHNFKKLS--TEGQS  190
CCGaV          108  LKVFDHQGFKPNEILKSLMVQARKNKVSKEDFKTDILMMCAISIIKGSI-----NEHNFKKLS--TEGQT  170
WCLaV_1        108  -RIFEYQGFNPDEILKSLIIAQRKNKISGDQFKTDILTLCAISIIKGSI-----NDHNFGKIS--KEGQD  169
WCLaV_2        105  LKIFEYQGFNPKAILISLMKSCDKNKIPKEQFKSDIITLCAISIIKGSI-----NSNNIKKVS--EEGQQ  167
UHBV            35  -GQIEYNGYDAGKLLQILKKKA-----EGRDFGKDLCYLLVMRYTRGTG-----FVRDVRKKIKTAAGGD   93
IWSV            35  -GQIDYNGYDAGKLLKILQDKS-----KNRDFGKDLCHLLVMRYTRGTG-----FVRDVRKKIKVAAGGE   93
RHBV            35  -GQIEYNGYDAGKLLQILKRKA-----EGRDFGKDLCYLLVMRYTRGTG-----FVRDVRKKIKTAAGGD   93
EHBV            33  -GQIEYNGYDAGKLLQILKKKA-----EGRDFGKDLGFLLVVRYTRGTG-----FVRDVRKKIKTAAGGD   91
RSV             39  -GQIEYAGYDAATLIGILKDKG------GDTLAKDMTMCITMRYVRGTG-----FVRDVTKKVKVAAGST   96
MSV             39  -DQIGYAGYDAATLIGILKDKG------GATLAQDVVKMIVMRYVRGTG-----FVKDVTKKTKATAGSE   96
RGSV            43  AAGLEYQAFNPEKILRKLIASS----TSLDDFVKDMRDLLVARYTRGTSFLFNAKNSIEKAKD--KKKAE  106
LLV             24  -TLFAYEGFNPEMIHSHFAKIMTEKGIGEMEFVNDMRALITLGAMKGNY-----TMKNAGKIS--EAGRT   85
Consensus_ss:        hhhhh    hhhhhhhhhhhh      hhhhhhhhhhhhhhhhh         hhhhhhhh   hhhhh
```

```
                                                *
GGDV             99  RIEELSKKYNIKIKKT---KNENSTLSNSTLTFTRSISVFP-YVASQLLATGACILEPNNCKFG-TDDLP  163
GMRV             99  RIEELSKKYNIKIKKS---KSEKMVLSNSTLTFTRSISVFP-YVASQLLATRSCEVEPNNCPFG-TDDLP  163
ARWV1            99  RIEELAKKYNIKMKKT---KKDKIALTNSTMTFTRSISVFP-YVASQLLSQSSCILEPTGCKFG-SDDLP  163
ARWV2            99  RIEELSKKYNIKIRKT---KNEKLVLSNSTLTFTRSISVFP-YVASQLLSTGSCVLEQNNCEFG-SDELP  163
UUKV             91  EFALLKARYQLVDKAK---EAADLTLSRIAIANAGLTCRILPQVVAHTAVTRSR----MES---LSADYP  150
SFNV             87  IVANLISVYQLKEGNP---GRDTITLSRVSAAFVPWTVQALRVLSESLPVSGTT----MDA--IAGVTYP  147
TOSV             87  IVANLISVYQLKEGNP---GRDTITLSRVSAAFVPWTVQALRVLSESLPVSGTT----MDA--IAGVTYP  147
RVFV             82  TVEALINKYKLKEGNP---SRDELTLSRVAAALAGWTCQALVVLSEWLPVTGTT----MDG---LSPAYP  141
SFTSV            82  RLMALQEKYGLVERAE---TRLSITPVRVAQSLPTWTCAAAAALKEYLPVGPAV----MNL---KVENYP  141
CiVA            191  TVIRLEKTYGIKRGSGRSEPANVVTISRIGATFPGKIIQLLV---ENKASSRKF----MGP--LKSHTLP  251
CCGaV           171  EVQRLEKTYGIKRGSGRNEPANVVTISRIGATFPGKIIQLFL---AGKVTARKF----IGP--FKSHTLP  231
WCLaV_1         170  AIVKLENSYGIKRGSGRGMNSTVVTVSRIAATFPGIVIQLLR---DNKVSARKF----IGP--LKSQTLP  230
WCLaV_2         168  EISRLETLYNIKRGGGRKERPDVITVSRIGATFPGKIVQLIH---AGKVQSRAF----MGP--FSSSSLP  228
UHBV             94  TAHEIVTHYGVVHSVG--DNADAITLGRLASLFPAVSMNIVRNVSTGAKLA-------VDSSDLGSSGLD  154
IWSV             94  TSHEIVTHYGIVQSVG--DNADAITLGRLAALFPNVSMTIVKSVSTGAKLA-------IDSADMGTSGID  154
RHBV             94  TAHEIVTHYGVVQSVG--DNADAITLGRLASLFPAVSMNIVRNVSTGAKLA-------VDSSDLGSSGLD  154
EHBV             92  TAHEIVTHYGVVQSVG--DNADAITLGRLASLFPAVSMSIVKNVSSGAKLA-------VDSSDLGTSGLD  152
RSV              97  EASTLVSRYGIVSSVG--TNANAITLGRLAQLFPNVSHEVVRQIS-GVKMA-------VDSSDLGLTGCD  156
MSV              97  EAAALVARYGLVSSVG--SNANAITLGRVAQLFPNVSFEVTKQFT-GLKMA-------IDSSDLSMSGTD  156
RGSV            107  AIQVLINRYGVKKNAG-DNAVDQATLGRISQVLAYMALRVALQITDYHKPIIPL-----RP--ISTVDIK  168
LLV              86  KADGLYKKYNMKQGSL-GGDKKAVILPRVLSAFPELTTKVV--LRTPPRDFGTR-----------TTRLP  141
Consensus ss:        hhhhhhhhh                     hhhhhhhhhhhhhhhhhhhhhhh

                              *
GGDV            164  AAFK--HSGFSSLIPNIAD----NNYCLVLFQCHVAYMISFGKKINPE---------NKDDDLTWYKKQT  218
GMRV            164  VAFK--HSGFSSLIPTG------NNYCLVLFQCHIAYMISFGKKINPE---------NKDDITVWYNKQK  216
ARWV1           164  AAFK--HSGFASLIPIG------NDYCSILFQCYLAYMISFGRKINPE---------NKEEDKVWYAKQR  216
ARWV2           164  SAFK--HSGFASLIPTG------NNYCTVLFQCHIAYMISFGKKIDPA---------NKDDVITWYNKQK  216
UUKV            151  VCMM--HNAFAGLIDETLP----EDSIKALVDAHRLYLLEFSRTINVKHRG-----MEAKEILDAND---  206
SFNV            148  RAMM--HPSFAGIIDLDLP----NGAGATIADAHGLFMIEFSKTINPSLRT-----KQANEVAATFE---  203
TOSV            148  RAMM--HPSFAGIIDLDLP----NGAGATIADAHGLFMIEFSKTINPSLRT-----KQANEVAATFE---  203
RVFV            142  RHMM--HPSFAGMVDPSLP----GDYLRAILDAHSLYLLQFSRVINPNLRG-----RTKEEVAATFT---  197
SFTSV           142  PEMM--CMAFGSLIPTAGVS---EATTKTLMEAYSLWQDAFTKTINVKMRG-----ASKTEVYNSFR---  198
CiVA            252  QFIR--HQGFAAVIPTTLA----ESTRMFLLDIVTAYSVDQSISISPNKK-------NKPDLASLYEKQR  308
CCGaV           232  QYVR--HQAFSAVIPSSLN----ERTKMYLLDIITAFSVDQSICISPNKK-------EKPDLSNLFEKQR  288
WCLaV_1         231  AVMR--HQAFAAVIPTALR----EKTRNFLLILITAFSVDQSINISPNKK-------EKPDVEGLFKLQE  287
WCLaV_2         229  PVMR--HQAFAAVIPRSMN----HKIKEFLLQLITAFSVDQSISINPNKK-------EKMDPVTTFSTQH  285
UHBV            155  LLLWDFVPQFITLDSVTAPYCSGKGMNIILFSLHVLHGALTTKKTMPDQKKKARNLVTDFELVKYTT---  221
IWSV            155  ILLWDFVPQFISLDSETAPFCNKKNANNVLLSLQILQGALTTKKTMPDQKKKLRNLITDYDFVKYTS---  221
RHBV            155  LLLWDFVPQFITLDSLTAPYCTGKGMNTILFSLHVLHGALTTKKTMPDQKKKARSLVTDFELVKYTT---  221
EHBV            153  ILLWDFVPQFITLDSVTAPYCTGKGMNVILFSLHVLHGALTTRKTMPDLKKKARSLVTDFELVKYTT---  219
RSV             157  NLLWDYVPQYIKLESETAPYCSTHSLSHILFVVHIIHSFQITKKTMPEGKKKERGLTKDIDMMKYTT---  223
MSV             157  SLLWDFVPQYITLDSSSAPYCTTKSVAHILFSIHVIHAFLVTKKTMPEAKKKERGLLKDIDIIKYTT---  223
RGSV            169  NAIIDVVPQFLYLKADQLDSK--TNSEAALYVIHLCYQVCVSERIMTKAQKDKHSVHTKSAMITHCM---  233
LLV             142  KYIK--NPVFPSLVPKTLQ----GDVAKTFLWLYTVYSAEQSLVIS-----------QEKDFNAAFTAQK  194
Consensus ss:        hhhh    hh              hhhhhhhhhhhhhhhhhhhhh  hhh         hhhhhhhhh

GGDV            219  QYSMAAWNNLSLYNNDFRIT----------------------AFGNLG-----------LDSH-------  248
GMRV            217  QFSFAAWNNVTLYNNEYRKK----------------------AFKNLG-----------LDSH-------  246
ARWV1           217  QYSYAAWSNLGLCTSDYRRS----------------------AFRNLG-----------LDSH-------  246
ARWV2           217  QYSIAAWNNMSLYDNDYRKT----------------------AFKNLG-----------LDSH-------  246
UUKV            207  SALQAGLA-SSFLTPSQKRA----------------------YLLSFK-----------LVDG-------  235
SFNV            204  KPNMAAMS-GRFFTREDKKK----------------------LLIAVG-----------IIDE-------  232
TOSV            204  KPNMAAMS-GRFFTREDKKK----------------------LLIAVG-----------IIDE-------  232
RVFV            198  QPMNAAVN-SNFISHEKRRE----------------------FLKAFG-----------LVDS-------  226
SFTSV           199  DPLHAAVN-SVFFPNDVRVK----------------------WLKAKG-----------ILGP-------  227
CiVA            309  SFI-QTTA-DNKYPSEIVRK----------------------KIFTTI-----------MIDY-------  336
CCGaV           289  AFV-QTTS-DAEYPPEIVRK----------------------KIFSTI-----------LLDY-------  316
WCLaV_1         288  NFIQVSAD-NIYPPESIRVK----------------------IFKSV------------ILNY-------  315
WCLaV_2         286  NYIQVIHD-GEYPPENIKKQ----------------------IFKTIS-----------INFD-------  314
UHBV            222  ELLVITCN-AKNLKAEKKTSYRESLMRPFQEGAGDALGFKETFWTSLA-----------KVSN-------  272
IWSV            222  ELMVITCS-AKNLTDEKKQTYRRKLVSAFQTDD---SGYKQEFWDALG-----------QVSV-------  269
RHBV            222  ELLVITCN-AKNLKVEKKTIYRESLMRPFQEGAGDALGFKETFWTSLA-----------KVST-------  272
EHBV            220  ELLVITCN-AKNLKAERKTAYRKSLMSSFQEGAGGDLGFKESFWTSLA-----------KVST-------  270
RSV             224  GLLVITCK-SKNLSDKKKEEGRKKVLDEFITNG----KVKTTIFDALA-----------GMSV-------  270
MSV             224  GLLVITCQ-SKNLNEAKKKSGRTKVCEPYCVNE----KFKESFLALLA-----------SFGK-------  270
RGSV            234  GFVNLAMD-NSSVVSDDKIA----------------------GRRMISGP-------WGLQETALDATGC  273
LLV             195  QFVTIAFN-SSVPDETTRIG----------------------MFKAKLEDLVQAISDFKDFTD-------  234
```

```
Consensus ss:        hhhhhhh       hhhhhh                      hhhhhh


GGDV            249  ------------GKYG----------ELYFKI----------VNNLRKSIKEGEVKPADLEPFIKEDKNL  286
GMRV            247  ------------EKYG----------ALYLKI----------VNNIRKAIKEEEVKPEDLAPYVKEGKIE  284
ARWV1           247  ------------TNYG----------AIYLKV----------ANNLRKSLGDPEVTIAYLETFVKSGQIE  284
ARWV2           247  ------------EKYG----------IVYMKV----------VNKLRKAINEEEVQLSDLAAFIKDGSIE  284
UUKV            236  ------------NGKV----------NKAVQQ----------AATVLRSLI-------------------  254
SFNV            233  ------------DLVL----------ASAVVR----------SAEKYRAKV---------GK--------  253
TOSV            233  ------------DLVL----------ASAVVR----------SAEKYRAKV---------GK--------  253
RVFV            227  ------------NGKP----------SAAVMA----------AAQAYKTAA-------------------  245
SFTSV           228  ------------DGVP----------SRAAEV----------AAAAYRNL--------------------  245
CiVA            337  ------------DSLL----------TTARALLKVIPDFTIVTKENFVSDI---------NSTHI-----  370
CCGaV           317  ------------DQLL----------STARAILKVFPEFVIVSKENFNADI---------NATHI-----  350
WCLaV_1         316  ------------DDLL----------TCARSISKFINDIIIPTRDEFMSDI---------SQL-------  347
WCLaV_2         315  ------------DLLV----------TAR-KIVALTGNLEIPSKDEFNSAV---------MSFN------  346
UHBV            273  ------------GCVRKMKKDAQTYLSERTSLLKVFVDNCNKAEAEVTEAI---------KAYFVTES--  319
IWSV            270  ------------GCVRKLKKDAQNYLKDRTTVLKVMVENCTKTEAEAIEAI---------KAFFAPEDV-  317
RHBV            273  ------------GCVRKMKKDAQAYLSERTPLLKVFVENCNKSEDEVAEAI---------KSYFVTGA--  319
EHBV            271  ------------GCVKKMKKDAQAYLSERTSLLKLFVENCNKTEAEVTEAI---------KSYFVTDA--  317
RSV             271  ------------NTISTYGNQTRLYLAQQSKLMKILAENTSKTATEVSGLV---------KEFFEDEAEG  319
MSV             271  ------------NVVCSYGTQVKQFLAEQCSLMKTIVDNSSKTQDEMKALI---------IEFFEEE---  316
RGSV            274  ACIIDVVDFCCRGHKV----------TDAVAP-----------VRLFRLAI---------ECI-KDTADL  312
LLV             235  ------------GEVP------------TNRS----------DARVARDAI---------TTL-------  254
Consensus_ss:                                  hhhhhh          hhhhhhhhh         h


GGDV            287  KW-----------  288
GMRV                 -------------
ARWV1           285  AKGSIS-------  290
ARWV2           285  QV-----------  286
UUKV                 -------------
SFNV                 -------------
TOSV                 -------------
RVFV                 -------------
SFTSV                -------------
CiVA                 -------------
CCGaV                -------------
WCLaV_1              -------------
WCLaV_2              -------------
UHBV                 -------------
IWSV                 -------------
RHBV                 -------------
EHBV                 -------------
RSV             320  ADD----------  322
MSV                  -------------
RGSV            313  KDAGVKLKTLVDK  325
LLV                  -------------
Consensus_ss:
```

Figure S3. Multiple sequence alignment of nucleocapsid proteins (NPs) encoded by grapevine Muscat rose virus (GMRV), grapevine Garan dmak virus (GGDV) and NPs encoded by viruses representative of several genera. The virus name and distance in amino acids from the N-terminus of the protein are shown on the left-hand side of each sequence. Consensus secondary structure (ss) elements, predicted by PROMALS3D, are reported at the bottom, with the helices indicated by “h”. Amino acids that fold in strands and helices are in blue and red, respectively. Green asterisk (*) indicates a tyrosine involved in RNA binding in RVFV and UUKM (Mottram et al., 2017; Raymond et al., 2012), while blue asterisk marks three positive charged amino acids predicted to be involved in the protein RNA binding activity in RVFV and RSV (Ferron et al., 2011; Lu et al., 2017). The grey asterisk denotes the phenylalanine residue shown to have an essential role for infectivity in RFVF (Mottram et al., 2017).
