# Supplementary material for: Two Novel Negative-Sense RNA Viruses Infecting Grapevine Are Members of a Newly Proposed Genus within the Family Phenuiviridae
Source: Viruses. 2019 Jul 26;11(8):685. doi: 10.3390/v11080685 (PMC6724010; doi:10.3390/v11080685)
Supplement: Supplementary file 1 [file viruses-11-00685-s001.zip › Figure S4.pdf]

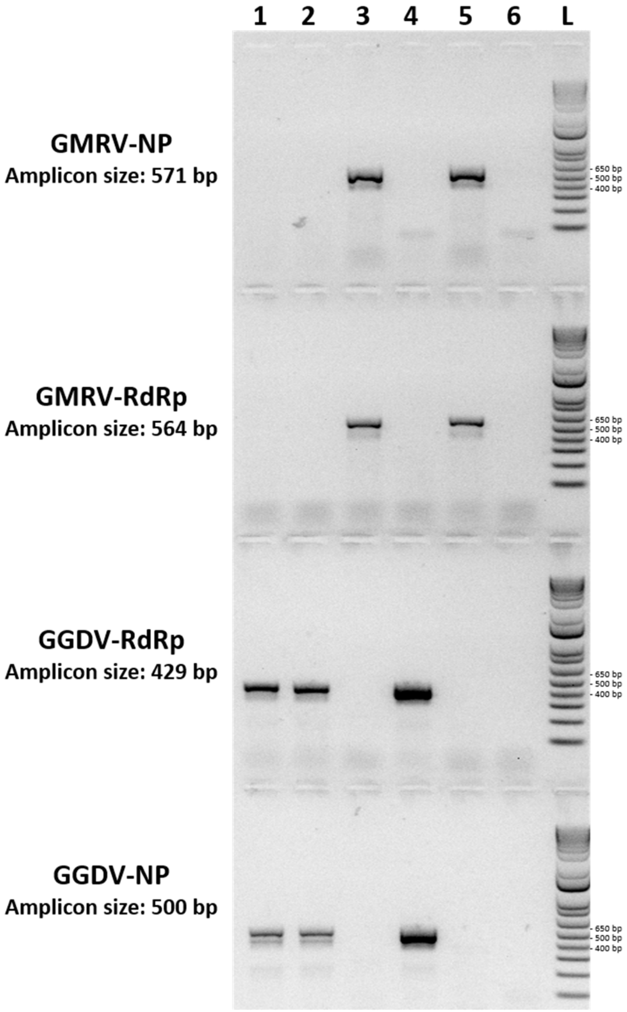

Figure S4. Detection of grapevine Muscat rose virus (GMRV) and grapevine Garan dmak virus (GGDV) in grapevine plants by reverse transcription PCR (RT-PCR). Lane 1, Alloued zeine; lane 2, Dizmar; lane 3, Chenin blanc; lane 4, Garan dmak; lane 5, Muscat rose; lane 6, negative control (water); lane L, 1 Kb Plus DNA Ladder. Detection by different RT-PCR assays (listed on the left side) targeting the RNA-dependent RNA polymerase (RdRp) and nucleocapsid protein (NP) of GMRV and GGDV.
