# Supplementary material for: Two Novel Negative-Sense RNA Viruses Infecting Grapevine Are Members of a Newly Proposed Genus within the Family Phenuiviridae
Source: Viruses. 2019 Jul 26;11(8):685. doi: 10.3390/v11080685 (PMC6724010; doi:10.3390/v11080685)
Supplement: Supplementary file 1 [file viruses-11-00685-s001.zip › Table S1.pdf]

Table S1. PCR primers used for the detection of novel negative sense-RNA viruses in grapevine.

Virus	Primer Name	Primer Sequence	Region	Expected Amplicon Size (bp)
Grapevine Garan dmak virus	GGDV-RDRP-F	5' GCATGATTGCCCTGACTAATGG 3'	RdRp	429
	GGDV-RDRP-R	5' GCAGCTGGTTTGGGAATTCA 3'		
	GGDV-NP-F	5' CTGATGACTTGCCTGCAGC 3'	Nucleocapsid protein	500
	GGDV-NP-R	5' TCCTTGGGCCATAAGGTTGC 3'		
Grapevine Muscat rose virus	GMRV-RDRP-F	5' TGACGAGGTAACTAAGTCTGC 3'	RdRp	564
	GMRV-RDRP-R	5' GTAGTTAAAGATCCCTCTCCAG 3'		
	GMRV-NP-F	5' GAGGCAATATAAGGGCTGA 3'	Nucleocapsid protein	571
	GMRV-NP-R	5' GGCTTTACTTCCTCTTCCTT 3'		
