# Supplementary material for: Two Novel Negative-Sense RNA Viruses Infecting Grapevine Are Members of a Newly Proposed Genus within the Family Phenuiviridae
Source: Viruses. 2019 Jul 26;11(8):685. doi: 10.3390/v11080685 (PMC6724010; doi:10.3390/v11080685)
Supplement: Supplementary file 1 [file viruses-11-00685-s001.zip › Table S2.pdf]

Table S2. High throughput sequencing (HTS) data generated from the Muscat rose and Garan dmak grapevines.

Sample	Pass Filter Reads	Yield	Average Read Length (nt)
Garan dmak	26,726,694	2,004,502,050	75
Muscat rose	172,311,649	13,095,685,324	75
