# Supplementary material for: Two Novel Negative-Sense RNA Viruses Infecting Grapevine Are Members of a Newly Proposed Genus within the Family Phenuiviridae
Source: Viruses. 2019 Jul 26;11(8):685. doi: 10.3390/v11080685 (PMC6724010; doi:10.3390/v11080685)
Supplement: Supplementary file 1 [file viruses-11-00685-s001.zip › Table S3.pdf]

Table S3. Nucleotide sequences identities between grapevine Muscat rose virus, grapevine Garan dmak virus and apple rubbery wood virus 2.

Pairwise identity (%)	Apple rubbery wood virus 2*		
	RNA 1 (RdRp)	RNA 2 (MP)	RNA 3 (NP)
Grapevine Muscat rose virus	60.85	77.06	73.38
Grapevine Garan dmak virus	56.28	62.25	58.62

RdRp, RNA-dependent RNA polymerase; MP, movement protein; NP, nucleocapsid protein.

*GenBank accessions of apple rubbery wood virus 2: RNA 1, MF062128; RNA 2, MF062145; RNA 3, MF062140.
